# Association of Impulsivity and Polymorphic MicroRNA-641 Target Sites in the SNAP-25 Gene

**DOI:** 10.1371/journal.pone.0084207

**Published:** 2013-12-31

**Authors:** Nóra Németh, Réka Kovács-Nagy, Anna Székely, Mária Sasvári-Székely, Zsolt Rónai

**Affiliations:** 1 Department of Medical Chemistry, Molecular Biology and Pathobiochemistry, Semmelweis University, Budapest, Hungary; 2 Institute of Psychology, Eotvos Lorand University, Budapest, Hungary; University of Wuerzburg, Germany

## Abstract

Impulsivity is a personality trait of high impact and is connected with several types of maladaptive behavior and psychiatric diseases, such as attention deficit hyperactivity disorder, alcohol and drug abuse, as well as pathological gambling and mood disorders. Polymorphic variants of the SNAP-25 gene emerged as putative genetic components of impulsivity, as SNAP-25 protein plays an important role in the central nervous system, and its SNPs are associated with several psychiatric disorders. In this study we aimed to investigate if polymorphisms in the regulatory regions of the SNAP-25 gene are in association with normal variability of impulsivity. Genotypes and haplotypes of two polymorphisms in the promoter (rs6077690 and rs6039769) and two SNPs in the 3′ UTR (rs3746544 and rs1051312) of the SNAP-25 gene were determined in a healthy Hungarian population (*N* = 901) using PCR–RFLP or real-time PCR in combination with sequence specific probes. Significant association was found between the T–T 3′ UTR haplotype and impulsivity, whereas no association could be detected with genotypes or haplotypes of the promoter loci. According to sequence alignment, the polymorphisms in the 3′ UTR of the gene alter the binding site of microRNA-641, which was analyzed by luciferase reporter system. It was observed that haplotypes altering one or two nucleotides in the binding site of the seed region of microRNA-641 significantly increased the amount of generated protein *in vitro*. These findings support the role of polymorphic SNAP-25 variants both at psychogenetic and molecular biological levels.

## Introduction

Impulsivity is a multidimensional personality trait characterized by action without planning and lack of consciousness to guide acts and behavior [1]. The phenotype is based on a complex neurochemical background, and genetic factors play a crucial role in its development, twin studies suggested that inheritance plays approximately 45% role in its determination [2]. There is a relatively broad spectrum of individual differences in healthy subjects regarding their impulsivity, therefore to summarize this trait in one specific definition has been a challenge for many decades. There is an agreement however that major deflection of impulsivity is one important component of Attention Deficit Hyperactivity Disorder (ADHD) together with impaired attention and/or hyperactivity [3].

Recent studies have focused mainly on the serotonergic [4] and dopaminergic [5] system investigating the genetic background of impulsivity, and our group has also previously shown the importance of the interaction between these two systems [6]. Further studies showed an association between impulsivity and a functional dinucleotide repeat polymorphism in the promoter region of NOS1 gene (exon 1f-VNTR) [7], [8], and the rs11624704 of the neurexin gene (NRXN) was also suggested to contribute to the background of impulsivity [9].

Impulsivity is one of the core features characterizing ADHD, thus, candidate genes shown to associate with ADHD might emerge as putative genetic components of impulsivity. A number of association studies as well as meta-analyses suggested the role of *DRD4*, *DRD5*, *HTR1B* and *SLC6A3* genes, reviewed by Faraone [10]. Moreover, several studies pointed out an association between two SNPs (rs3746544 and rs1051312) in the 3′ UTR of the *SNAP-25* gene and ADHD [11], [12], [13], and the role of the former polymorphism was also confirmed in a comprehensive meta-analysis [14]. Genetic variants of *SNAP-25* including promoter polymorphisms [10], [12], [15], have already been investigated as putative risk factors of other psychiatric disorders as well [16], [17], but it has not yet been studied in the background of impulsivity in a non-clinical sample.

SNAP-25 (synaptosomal-associated protein, 25 kDa) plays a crucial role in the central nervous system, being one essential component of the SNARE (soluble N-ethylmaleimide-sensitive factor attachment protein receptors) complex and contributes to exocytosis by targeting and fusion of vesicles to the cell membrane [18]. There are two SNPs (rs3746544 and rs1051312) in the 3′ UTR of the *SNAP-25* gene with special interest, we previously demonstrated by *in silico* sequence analysis that they are localized in the putative target site of miR-641 [19].

As microRNAs are known to play a role as translational regulators of protein synthesis, SNPs located either in the coding sequences of microRNAs or in their binding sites might cause an imbalance of this regulation. SNPs have been described in genomic regions encoding miRNAs [20], the rs1625579 polymorphism is located in the coding region of miR-137, which has been shown to target the *ZNF804A*, a candidate gene of schizophrenia [21]. However, more attention have been focused on the significantly higher number of SNPs that are present in the 3′ UTR of target genes altering the binding region of a miRNA to its specific target mRNA [20]. Polymorphisms in the microRNA-binding sites have been suggested to contribute to the genetic background of various diseases, such as coronary heart disease [22], different types of cancer [23], [24] or ADHD and other co-morbid psychiatric illnesses [12], [25]. A polymorphic variant (rs13212041) in the *HTR1B* gene 3′ UTR was demonstrated to influence miR-96 binding and to be associated with conduct-disorder phenotype in a sample of 359 students [26].

Here we present data on the functional effect of rs3746544 and rs1051312 SNPs and their haplotypes on miR-641 regulated reporter gene expression in cell culture. We also aimed to carry out an association study to analyze whether either these loci or the polymorphic variants of the *SNAP-25* promoter (rs6077690 AT and rs6039769 AC), studied so far in psychiatric disorders, contribute to the genetic background of impulsivity in a non-clinical sample.

## Methods

### Ethics Statement, Participants

901 healthy Hungarian young adults participated in the study. Selection criteria included no past or present psychiatric history (based on self-report). Before providing buccal samples for genetic analysis, participants signed written informed consent. The study protocol was approved by the Scientific and Research Ethics Committee of the Medical Research Council (ETT TUKEB). The mean age of the investigated population was 21.3 (±3.3) years, 45.1% were males and 54.9% were females.

### Phenotype analysis

Hungarian version of the 11^th^ revised Barratt Impulsiveness Scale [27] was used to measure impulsivity. The questionnaire was originally published by Barratt. Translation into Hungarian was carried out by a “forward-backward” procedure [6], Cronbach alpha value for the total score of the scale was 0.808.

The Barratt Scale is a self-reporting measure widely used both by clinicians and in research settings. It consists of 30 items regarding acting and thinking in different situations. These statements are asked to be rated by the participant on a four-point-scale, reaching from “occasionally” to “always”. The highest theoretically possible total score is 120 and impulsivity is considered “normal” in the range from 52 to 71.

### DNA isolation

DNA purification was initiated by incubating the buccal samples at 56°C overnight in 0.2 mg/ml Proteinase K cell lysis buffer. It was followed by protein denaturation using saturated NaCl solution. Finally, DNA was precipitated using isopropanol and ethanol by standard procedures and DNA pellet was resuspended in 100 µl 0.5× TE (1× TE: 10 mM Tris pH = 8, 1 mM EDTA) buffer. Concentration of each DNA-sample was measured by Varioscan Flash spectral scanning multimode reader.

### Genotyping of SNAP-25 SNPs

The promoter SNP rs6039769 was genotyped by the C__29497348_10 (Life Technologies) pre-designed primer- and TaqMan probe-set. A 7300 Real-Time PCR System (Life Technologies) was employed to detect the FAM and VIC signals corresponding to the C and A alleles, respectively.

Genotypes of rs6077690 promoter SNP were determined by PCR-RFLP. Flanking region of the polymorphic site was amplified using the 5′ ATG TCA GTG TGG GGC ATC 3′ sense and 5′ AGG CAT GTT GCT GAA ATT TGT T 3′ antisense primers. The Qiagen HotStarTaq DNA-polymerase system was applied for PCR amplification, the reactions were carried out in a total volume of 10 µL containing 1 µM sense and antisense primers, 0.2 mM of each deoxyribonucleotide-triphosphate, 0.25 U HotStarTaq DNA-polymerase together with 1× buffer and 1× Q-solution and approximately 4 ng genomic DNA sample. Thermocycle was initiated by 15 min at 95°C initial denaturation and polymerase activation. It was followed by 40 cycles of 1 min denaturation at 94°C, 30 sec annealing at 63°C and 1 min extension at 72°C. The last step of the PCR was a final extension at 72°C for 10 minutes, amplicons were then kept at 8°C for downstream processing. In the next step PCR-products were digested by *Tsp*I 509 restriction endonuclease. Two non-specific recognition sites were incorporated in the amplicons to verify optimal conditions of digestion. Reactions were carried out accoriding to manufacturer's instructions. A 301- and a 110-bp-long product (together with the two control fragments) were generated in the presence of the A allele, whereas the 411-bp long fragment could be seen in case of the T allele. Digestion pattern was analyzed by traditional submarine gel electrophoresis. Call rate of genotyping was 97%.

### Direct haplotyping of SNAP-25 3′ UTR SNPs

As the two SNPs (rs3746544 and rs1051312) in the 3′ UTR are separated by only 3 basepairs from each other, it was possible to identify haplotypes directly in each individual sample using the published “double-tube” method [19]. In summary, the method is based on the application of haplotype-specific TaqMan probes in a real-time polymerase chain reaction. Two simultaneous analyses contained the four different probes corresponding to the four haplotypes labeled by FAM and VIC, respectively. Amplification and data-collection were done by a 7300 Real-Time PCR System (Life Technologies). Call rate was 98%. Individual genotypes of rs3746544 and rs1051312 were deduced from haplotype data.

### Haplotype analysis of SNAP-25 promoter SNPs

SNPs in the promoter were about 1.5 kb apart from each other which provided rather limited possibilities for simultaneous analysis and thus direct haplotype determination. Therefore individual haplotypes for these SNPs were calculated from genotype data. Haplotype was ambiguous only in case of double heterozygote individuals (rs6077690 AT and rs6039769 AC; i.e. either A–A/T–C or A–C/T–A). Linkage disequilibrium analysis, however, revealed that the frequency of the T–A haplotype was as low as 0.6%. As a total of 103 double heterozygotes were identified in our sample, approximately 1 participant was mathematically expected to possess the rare T–A haplotype (i.e. A–C/T–A), which was neglected. Using this assumption, the haplotypes of double heterozygotes were also able to be identified from the genotypes, as these haplotypes were considered to be A–A/T–C. To confirm this approach, haplotypes were also reconstructed by Phase 2.1, which provided the same result for each sample [28].

### Plasmid constructs

The entire 3′ UTR region of the human *SNAP-25* gene was cloned behind the firefly luciferase gene at the multicloning site of the pMIR-REPORT Luciferase miRNA Expression Reporter Vector (Life Technologies), using the 5′ TGT AAT ***GAG CTC*** CTG GGA AGT GGT TAA GTG T 3′ sense and antisense 5′ CCC GAC ***AAG CTT*** AAA CTA GCT ACA AAA TGT CAA TCA 3′ primers. Bold italic letters show the recognition sites of *Sac* I and *Hind* III restriction endonucleases in the sense and antisense primers, respectively. Genomic DNA possessing a TT haplotype was applied to amplify the *SNAP-25* 3′ UTR, constructs with the GC, GT and TC haplotypes were subcloned by QuickChange Lightning Site-Directed Mutagenesis kit (Agilent Technologies). All four constructs were verified by direct sequencing. Another pMIR construct was used as internal control, which contained an insert with same length but different sequence and most importantly missing the binding site of the analyzed miR-641.

### Cell culture and transient transfections

Human embryonic kidney (HEK) 293 cells (purchased from Sigma-Aldrich Ltd. Budapest, Hungary) were cultured in 24-well tissue culture plates in DMEM supplemented with 10% fetal bovine serum and 1% penicillin solution at 37°C in a humidified atmosphere containing 5% CO_2_. After optimization experiments 0.05 µg of the pMIR reporter constructs, 5 pmol miR641 and 0.2 µg *β*-galactosidase constructs were co-transfected in a reaction mixture containing 2.5 µl Lipofectamin and 60 µl OptiMem.

Cells were incubated at 37°C after transfection.

### Luciferase and β-galactosidase assay

Medium was removed 36 hours after transfection and cells were washed twice in phosphate buffered saline, and the cell extracts were suspended in 100 µL 250 mM Tris-HCl buffer.

Cell lysis was carried out by three consecutive freeze-thaw cycles, lysate was centrifuged (13000 rpm for 15 minutes at 4°C) and the supernatans were collected for luciferase and *β*-galactosidase activity measurements.

Luciferase activity was detected by adding 60 µL of luciferin reagent to 12 µL sample, *β*-galactosidase activity was measured by adding a reaction mixture containing 33 µL ONPG solution (ortho-nitrophenil-*β*-galactoside) to 20 µL sample, respectively. Fluorescence and luminescence values were measured using the Varioscan Flash spectral scanning multimode reader (ThermoScientific). Analyses were carried out in triplicates.

### Statistical analysis

Raw data of luciferase enzyme activities were normalized to the *β*-galactosidase levels for each sample. Statistical analysis was performed with the Tukey-Kramer multiple comparisons test. P values lower than 0.05 were considered to be statistically significant. *D*′ and *R* ^2^ measures of linkage disequilibrium were assessed using HaploView v4.2.

Genetic association analyses were carried out using SPSS 19.0 for Windows. Chi-square analyses were applied to test if genotype frequency distributions corresponded to Hardy–Weinberg-equilibrium. Independent-Samples t-test was used to test genetic associations by one way analyses of covariance (ANCOVA) assuming a bi-allelic inheritance model and co-dominant inheritance.

## Results

### Genotype and haplotype frequencies of SNAP-25 promoter and 3′ UTR region in healthy participants of European descent

**Figure 1:**
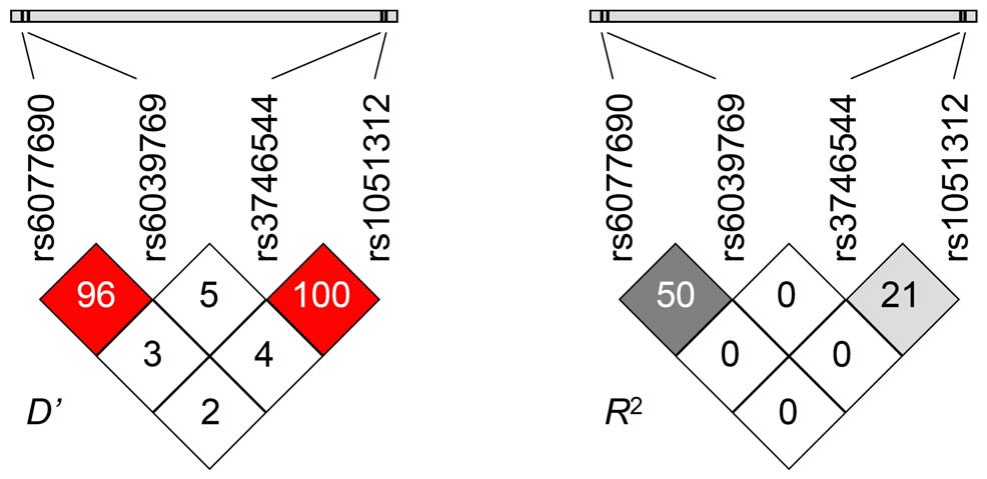
Linkage disequilibrium analysis of the two promoter (rs6077690 AT and rs6039769 AC) and two 3′ UTR (rs3746544 GT and rs1051312 CG) SNPs. Left panel: Lewontin′s *D*′ values, right panel: *R* ^2^ measure of LD. High *D*′ together with relatively low *R* ^2^ values in the promoter as well as in the 3′ UTR region suggest a partial linkage disequilibrium characterized by the decreased frequency of one haplotype combination compared to expected data. No LD could be observed between the promoter and the 3′ UTR regions.

Allele- and genotype distribution together with haplotype frequencies of two promoter polymorphisms (rs6039769 and rs6077690) as well as those of the two SNPs in the 3′ UTR (rs3746544 and rs1051312) of the *SNAP25* gene were determined in a healthy Hungarian population (*N* = 901). Genotype distributions were in Hardy–Weinberg-equilibrium for each SNP, minor allele frequencies varied between 0.255 and 0.417 (*Table 1*). As the two SNPs of the 3′ UTR were in close proximity (see *Table 1* for genomic localization), a direct haplotype determination was possible to perform for each individual DNA samples using haplotype-specific TaqMan probes, and genotypes were deduced from haplotype data. On the other hand, the two SNPs of the promoter region were genotyped individually as their greater distance did not allow a direct haplotype analysis, and haplotype frequencies were estimated by calculation. Analysis of the 3′ UTR haplotypes confirmed our previous result [19], that the G–C haplotype did not occur at all in the population. Interestingly a similar situation was observed regarding the promoter haplotypes. Although the T–A haplotype was not absent completely, its frequency (0.006) was much lower than expected (0.161). This resulted in a special type of linkage disequilibrium characterized by a high Lewontin's *D*′ value, together with a relatively low *R* ^2^. On the other hand absolutely no linkage disequilibrium could be observed between the promoter and the 3′ UTR region (*Figure 1*).

**Table 1:** Genotype and haplotype frequencies of the assessed SNPs in the *SNAP-25* gene.

SNP	Position on chromosome 20	Hardy–Weinberg *p*	Minor allele and its frequency (MAF)
rs6077690 AT	10,197,461	0.8899	A: 0.417
rs6039769 AC	10,198,954	0.4417	A: 0.276
rs3746544 GT	10,287,084	0.7394	G: 0.391
rs1051312 CG	10,287,088	0.1909	C: 0.255
rs6077690 AT –	rs6039769 AC	rs3746544 GT –	rs1051312 CG
Haplotype	Frequency	Haplotype	Frequency
TC	0.579	GT	0.391
AA	0.269	TT	0.354
AC	0.146	TC	0.255
TA	0.006	GC	0

### Association analysis of SNAP-25 SNPs and impulsivity

As a first step, a single marker analysis was used by comparing the average impulsivity scores of participants in the different genotype categories (Table 2). No association could be detected with the promoter SNPs. On the other hand, one of the 3′ UTR (rs1051312) polymorphism showed a nominal association with Barratt-scores of impulsivity (*p* = 0.042) which disappeared after Bonferroni correction for multiple test (4 SNPs: p<0.0125).

**Table 2:** Association analysis between the *SNAP-25* SNPs and impulsivity.

*SNP*	*Genotype*	*N*	*Mean*	*STD*	*P*
	GG	134	59.86	9.4	
rs3746544	GT	393	59.30	10.0	0.335
	TT	374	58.56	9.1	
	CC	49	62.39	9.2	
rs1051312	CT	326	59.02	9.6	**0.042**
	TT	526	58.80	9.5	
	AA	17	63.53	9.0	
rs6039769	AC	80	62.89	10.0	0.934
	CC	106	63.38	9.7	
	AA	38	64.79	8.9	
rs6077690	AT	95	62.26	10.3	0.411
	TT	69	62.97	9.7	

*N*: number of individuals possessing the given genotype, *STD*: standard deviation, *P*: level of statistical significance.

As a second step, we applied a haplotype-based allele-wise analysis for the promoter and the 3′ UTR SNPs, separately. No association was found between the estimated promoter haplotypes and total impulsivity scores (data not shown). It is important to note that the 3′ UTR haplotypes were not estimated but determined in each subject individually by molecular methods (see the “Methods” section). It was observed, that individuals possessing the T–T haplotype of the rs3746544 and rs1051312 SNPs achieved a lower total Barratt-score (58.24) compared to participants without this haplotype (59.63), and this effect was significant either calculating for the three haplotype categories (*p* = 0.009), or opposing the T–T haplotype (associating with lower impulsivity scores) to the others (p = 0.003). In other words, the 3′ UTR haplotypes of the *SNAP-25* gene had a more pronounced effect on the measured phenotype than the contributing SNPs, separately.

### Functional analysis of SNAP-25 3′ UTR haplotypes

**Figure 2:**
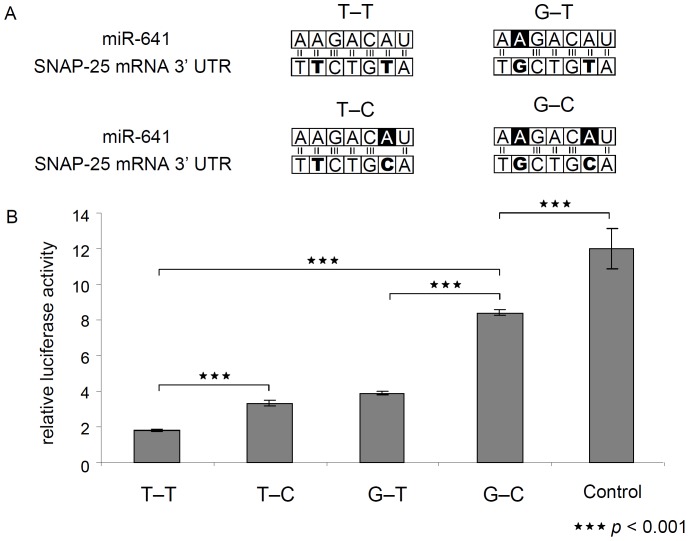
Effect of the two 3′ UTR (rs3746544 GT and rs1051312 CG) SNPs on miR-641 binding. *A* Sequence alignment of the seed region of miR-641 and the corresponding *SNAP-25* 3′ UTR region. Bold letters indicate the position of the two polymorphisms, white letters in black background symbolize the mismatches caused by the SNPs. *B* Normalized luciferase activities of reporter constructs containing the entire *SNAP-25* 3′ UTR with the four different haplotypes as well as of a control construct containing an insert with identical length, however lacking any binding site of miR-641. See text for details.

Based on the results of the association study, the 3′ UTR haplotypes showing an association with impulsivity were subjected to *in vitro* functional analysis. According to sequence alignment analysis both SNPs (rs3746544 and rs1051312) in the 3′ UTR are supposed to alter duplex formation of miR-641 and SNAP-25 mRNA, as the two polymorphisms are localized in the target site of the seed region of the miRNA-641 (*Figure 2A*). To study the molecular effect of the target sequence variants, pMIR-REPORT luciferase constructs containing the complete 3′ UTR region with the different haplotype variants were analyzed. This included the G–C haplotype as well, although it could not be detected in our investigated population. The lowest relative luciferase activity could be observed in case of the T–T haplotype, which generates a perfect target sequence for the seed region of miR-641. A single nucleotide change of the target sequence in constructs with the G–T and T–C haplotypes resulted in a 1.8 and a 2.1-fold elevation of relative luciferase activity, respectively. Although the single nucleotide change elevated significantly the reporter activity, there was no difference between G–T and T–C haplotypes, suggesting that a single base change alters significantly the binding of miR-641, but the position of the SNP within the seed region does not play a crucial role in this system. The G–C haplotype resulted in two mismatches in the target region of miR-641, and caused a 4.6-fold higher luciferase activity compared to the T–T form (*Figure 2B*), demonstrating that two mismatches in the target sequence have a more pronounced effect on miRNA function than one mismatch. The control construct had the same length, but an independent sequence without any binding site of miR-641. As expected, the corresponding luciferase activity was significantly higher than that of any of the constructs containing the 3′ UTR of the *SNAP-25* gene.

## Discussion

Recent studies have demonstrated that regulation of protein synthesis is much more complex than was earlier predicted. One major component of this network is the system of micro-RNAs (miRNAs) demonstrated by the fact that as high as 5% of the genes are predicted to encode miRNAs. It is also noteworthy that at least 30% of protein coding genes are suggested to be regulated by miRNAs [29], while this number could reach even 60% according to other estimations [30]. Alterations in miRNA profiles have been shown in the background of several illnesses, such as cancers, autoimmune diseases or cardiovascular disorders [31]. MiRNA profiling can be a useful tool in tumor diagnostics [32], and several miRNA-based therapeutic protocols have been elaborated. For example a miR-122 inhibitor was shown to decrease the amount of hepatitis C virus RNA, whereas a modulator of miR-208 level seemed to be effective against cardiac hypertrophy [33].

As a single miRNA might regulate a set of target genes, or the 3′ UTR of a specific mRNA might bind a number of miRNAs, the translational regulation by microRNAs is a complex system. Interestingly, as few as 20 SNPs have been identified in genomic regions coding for miRNA seed sequences. On the other hand, more than a hundred thousand SNPs are predicted to change miRNA target sites, but less than 1% of these functional variants have been validated experimentally [30].

The rs3746544 and rs1051312 SNPs in the 3′ UTR region of the *SNAP-25* gene have been thoroughly investigated as possible risk factors of ADHD [34], [35], [36] or bipolar disorder [12], however the molecular function of these variants has not yet been studied. Thus the question has been raised if these SNPs are genetic markers or causal polymorphisms [14], and according to our knowledge, our study is the first to analyze the effect of these SNPs on miRNA binding efficiency. Both studied SNPs are localized in the binding region of miR-641 in a close proximity of each other. Here we demonstrated that a single mismatch in the 3′ UTR of the SNAP-25 gene caused by any of the two SNPs resulted in about 2-fold reporter activity, while the rs3746544 G–rs1051312 C haplotype possessing two mismatches in the miR-641 target site led to a more than 4-fold elevation in the luciferase level. These results confirm the molecular functional role of rs3746544 and rs1051312 haplotypes in the SNAP-25 gene and are in agreement with the results of previous studies demonstrating the importance of optimal SNAP-25 level. The coloboma mouse, which is the animal model of ADHD has SNAP-25 deficiency and changes in SNAP-25 expression were shown to play a role in altered neuronal function [37]. It was also demonstrated, that atomoxetine which is an orally administered medicine used for the treatment of ADHD resulted in the significant up-regulation of SNAP-25 both on mRNA and protein level [38].

Here we aimed to investigate the impulsive behavior of a healthy population of European descent, and found a nominally significant association (p = 0.042) between rs1051312 and impulsivity which did not survive the Bonferroni correction for multiple testing (p<0.0125 for 4 SNPs). If, however, the 3′ UTR haplotypes were applied in the association study instead of the SNPs, lower impulsivity scores were observed in the presence of the T–T haplotype of the rs3746544 and rs1051312 SNPs as compared to the other haplotypes (p = 0.003). These results are in good agreement with our data obtained by the molecular analysis where the lowest reporter activity was measured in the presence of the 3′ UTR with T–T haplotype. Our findings are also in agreement with family studies, which showed association between the rs3746544G [39] and the rs1051312C alleles [40], although these results were not significant.

On the other hand the T allele of both SNPs was also shown to be the risk factor of ADHD. It is also notable, however, that the association had a modest statistical significance [10] and odds ratio was 1.15 and 1.06 for the rs3746544 and rs1051312 SNPs, respectively [14]. This shows that SNAP-25 is only one of the numerous genetic components of this phenotype. Taking into consideration that our study investigated impulsivity instead of ADHD in a healthy population, all data support, that SNAP-25 is one of the several genetic factors of impulsivity and related psychiatric disorders. Interestingly the haplotype with double mismatch (G–C) in the miRNA target site of SNAP-25 gene was completely undetectable in our healthy population of European descent (Hungarian) according to our previous [19] and current studies. It is important to note that haplotypes were not estimated but individually measured by a direct molecular haplotype analysis method developed earlier [19], [41]. A theoretically possible explanation of the absence of the G–C haplotype in our healthy volunteer population could be the more severe effect of this haplotype on SNAP-25 gene expression leading to pathophysiological consequences. Alternatively, the missing haplotype might be explained by the evolutionally young age of one of the SNPs, therefore recombinant haplotypes of these 3′ UTR SNPs, could not spread out in the population yet.

Although the putative biological effect of SNPs in regulatory regions both in 5′ or 3′ region is the modulation of protein level by different mechanisms, interestingly, much less data are available about the role of 5′ polymorphisms of the SNAP-25 gene. The rs6039769 SNP was shown to be in association with early-onset bipolar disorder [15], however no association could be found between ADHD and this locus [13]. On the other hand, the other polymorphism, located approximately 2 kb 5′ from the transcriptional start site (rs6077690) was demonstrated to be in association with ADHD [13]. Analysis of the two promoter SNPs and their haplotypes did not reveal any association between the polymorphisms and impulsivity. In conclusion, our results confirmed the findings of previous studies investigating SNAP-25 and ADHD. Our study focused on a healthy population, but even in this setting an association could be observed between the haplotypes of the 3′ UTR SNPs (rs3746544 and rs1051312) and normal individual variability of impulsivity. *In vitro* functional analyses suggested that these loci are miR-SNPs altering the binding efficiency of miR641 in a luciferase reporter system.
